# Uterine Rupture With Placenta Percreta Following Multiple Adenomyomectomies

**DOI:** 10.7759/cureus.34852

**Published:** 2023-02-11

**Authors:** Manabu Ogoyama, Kazuki Yamamoto, Hirotada Suzuki, Hironori Takahashi, Hiroyuki Fujiwara

**Affiliations:** 1 Obstetrics and Gynecology, Jichi Medical University, Shimotsuke, JPN

**Keywords:** pregnancy, cesarean hysterectomy, placenta percreta, uterine rupture, adenomyomectomy

## Abstract

Pregnancy following adenomyomectomy is challenging because uterine rupture or placenta accreta spectrum (PAS) is more likely to occur; however, optimal management has not yet been established. We herein present a case of uterine rupture with placenta percreta in a pregnant woman who underwent adenomyomectomy twice before pregnancy. Magnetic resonance imaging (MRI) was performed in the second trimester and imminent uterine rupture concomitant with PAS was suspected. The patient was immediately admitted to hospital for careful management. Although failed tocolysis forced delivery at 29 weeks of gestation, managed hospitalization allowed cesarean hysterectomy to be performed uneventfully. Extensive PAS was proven pathologically in the removed uterus. Pregnancies following multiple adenomyomectomies are considered to be high-risk. Therefore, a sufficient explanation of the risks associated with future pregnancies is needed, particularly following second adenomyomectomy.

## Introduction

Adenomyomectomy is one of the effective treatments for adenomyosis. Although dysmenorrhea and/or severe anemia may markedly improve after this procedure, the risk of uterine rupture in subsequent pregnancies is high [1-3]. Uterine rupture associated with a history of adenomyomectomy before pregnancy has two features as follows: it occurs at various stages of gestation [4-6] and is often complicated with placenta accreta spectrum (PAS), particularly placenta percreta. Uterine rupture with or without PAS occurring has been reported in pregnant women with a history of adenomyomectomy [3,7,8]. Obstetricians need to consider maternal symptoms, including uterine contractions and abdominal pain, to detect uterine rupture and/or PAS. Most cases achieve a good outcome for both mother and child with the above-described cautious management [2-4,7,9]. However, since uterine rupture is rare, risk factors for uterine rupture in pregnant women with a history of adenomyomectomy before pregnancy currently remain unclear. Furthermore, the optimal management of pregnant women following adenomyomectomy has not yet been established. We herein present a case of uterine rupture with placenta percreta in a pregnant woman who underwent adenomyomectomy twice before pregnancy. Magnetic resonance imaging (MRI) was performed in the second trimester and threatened uterine rupture concomitant with PAS before delivery was strongly suspected.

## Case presentation

A 40-year-old primiparous Japanese woman following in vitro fertilization was referred to our institution, which is a perinatal medical center, at 10 weeks of gestation because she had undergone adenomyomectomy twice before pregnancy. She was complicated by asymptomatic cholelithiasis. At 31 years of age, she underwent laparotomic adenomyomectomy for the first time and right ovarian endometrial cystectomy with severe dysmenorrheal symptoms (the weight of enucleated adenomyotic tissue was 104 g) at other institution. Since dysmenorrheal symptoms recurred, the patient underwent laparotomic adenomyomectomy again and bilateral endometrial cystectomy at the same institution at 38 years of age (the weight of enucleated adenomyotic tissue was 32 g). Adenomyotic tissue occupying the entire posterior wall of the uterus was removed each time, and the remaining anterior myometrium wall was used to form the uterus. The patient conceived 26 months after the second adenomyomectomy.

**Figure 1:**
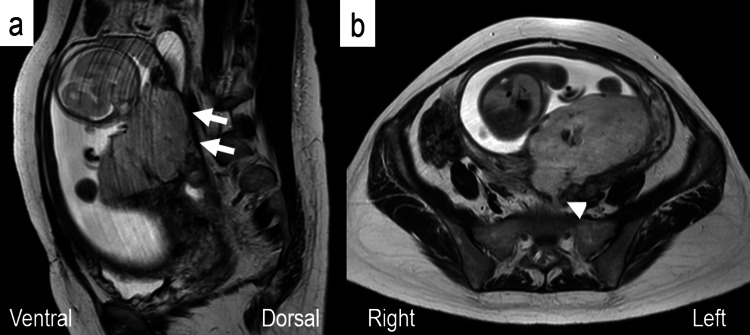
MRI findings at 22 weeks of gestation. (a) T2-weighted MRI revealed that the uterine wall from the bottom to the posterior wall of the uterus was thinning and bulging outward (arrows). (b) A part of the maternal side of the placenta was exposed outside the posterior uterine wall, suggesting the presence of placenta percreta (arrowheads).

She was admitted to our institute for the treatment of hyperemesis gravidarum from 10^2/7^ to 11^2/7^ weeks of gestation. Transvaginal ultrasound revealed a subchorionic hematoma (SCH) of 28×16 mm near the endocervical os. SCH extended up to 56 mm in length; however, abdominal pain was not observed at 13 weeks of gestation. Genital bleeding gradually stopped and SCH also shrank. Since the adenomyomectomy scar was present uterine posterior and the placenta was located there, the presence of PAS should be ruled out. However, ultrasound did not clearly show the presence or absence of PAS. MRI was performed at 22 weeks of gestation to evaluate the condition of the uterine wall and placenta because the patient was at high risk of uterine rupture. MRI revealed that the uterine wall was bulging outward from the bottom to the posterior wall of the uterus, suggesting the presence of PAS (Figures 1a, 1b). Furthermore, the posterior wall of the uterus strongly adhered to the colon. Although she had no abdominal pain or bleeding, the patient was considered to be in a state of threatened uterine rupture and, thus, was admitted to hospital for careful management from 23^0/7^ weeks of gestation. We told her and her husband that we would perform hysterectomy at delivery due to placenta percreta, and we obtained their consent.

**Figure 2:**
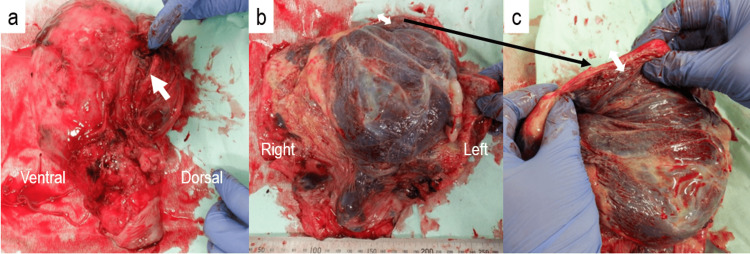
The findings of the removed uterus and placenta. (a) A part of the maternal side of the placenta was exposed outside the posterior uterine wall (arrow). (b and c) The majority of the placenta was adherent to the left posterior side of the uterine fundus and the uterine wall at this location was thinning.

She was managed with prophylactic tocolysis by ritodrine hydrochloride and bed rest; however, uterine contractions and shortening of the cervix (cervical length: 25 mm) were observed at 26 weeks of gestation. Tocolysis was enhanced by increasing ritodrine hydrochloride. Transabdominal ultrasound failed to identify the myometrium of the left side of the uterine fundus at which the placenta was located and showed multiple placental lacunae with hypervascularity. Although the patient was stable with tocolysis and bed rest, the preterm premature rupture of membranes (pPROM) occurred at 29^1/7^ weeks of gestation. The fetus was in breech presentation and uterine contractions were increasing; therefore, an emergency cesarean section was performed under spinal anesthesia on the same day, yielding a female infant (1353 g, APGAR score: 7/8 {1/5 min}, umbilical artery pH: 7.408, base excess: -2.9 mmol/L). The posterior uterine wall was very thin and bulging outward with extensive adhesion of the mesentery of the sigmoid colon. In addition, a part of the maternal side of the placenta was exposed outside the posterior uterine wall. We considered it difficult to preserve the uterus. Since the placenta was partially detached and external bleeding had increased, a total hysterectomy was performed after the rapid dissection of adhesions between the uterus and sigmoid colon under general anesthesia. Intraoperative blood loss was 6060 mL and allogeneic blood transfusion (20 units {U} of red blood cells and 14 U of fresh frozen plasma {FFP}) was performed. The patient was admitted to the intensive care unit (ICU) for postoperative systemic management. She was transfused with an additional 4 U of FFP in the ICU and did not develop disseminated intravascular coagulation; therefore, she was returned to a maternity ward on the second postoperative day. The removed uterus showed that more than half of the placenta adhered to the left posterior side of the uterine fundus (Figures 2a-2c). Pathological findings revealed that most of the adhered part was placenta increta, while the remainder was placenta percreta. The maternal postoperative course was uneventful, and she was discharged on postoperative day 11. The neonate was admitted to the neonatal ICU. No obvious malformations were observed in the neonate. Although the neonate was incubated and managed for transient tachypnea of the newborn, the subsequent course was uneventful.

## Discussion

This is the first detailed case report of pregnancy following multiple adenomyomectomies. MRI was performed at 22 weeks of gestation, which revealed the thinning and outward bulging of the uterine wall, leading to the diagnosis of imminent uterine rupture. The patient was immediately admitted to hospital for careful management to prolong the pregnancy period and prepare for delivery.

Adenomyomectomy performed before pregnancy increases the risk of a number of adverse events in the subsequent pregnant women, including uterine rupture. In adenomyomectomy, when adenomyotic tissue with an extensive, diffuse, and complex distribution within the normal uterine myometrium is resected as much as possible, the uterus is reformed with the remaining normal myometrium [2,10]. Therefore, the capacity of the uterus decreases and its tolerance for uterine contents with pregnancy is reduced. Furthermore, due to multiple suture points, the remaining uterus that is formed may be fragile. Numerous cases of uterine rupture after adenomyomectomy have been reported to date [1,2,4-6,11-13]. In contrast to myomectomy, uterine rupture after adenomyomectomy may occur at any time of pregnancy [4-6]. Furthermore, adenomyomectomy increases the risk of PAS in the subsequent pregnancy. When trying to resect as much adenomyotic tissue as possible, the endometrium often has to be opened. Hence, the risk of PAS will increase in the subsequent pregnancy. Therefore, the endometrium is missing, inducing excessive trophoblast invasion into the myometrium in subsequent pregnancies. If the fallopian tubes are removed with the resection of sufficient adenomyotic tissue, assisted reproductive technology is required for subsequent pregnancies. This may be another factor that increases the risk of PAS. Moreover, if total hysterectomy is required due to PAS with massive bleeding, adhesions between the uterus and surrounding tissues increase the difficulty of this procedure. When partial detachment of the placenta occurs concomitantly with PAS, the uterus needs to be removed as soon as possible due to continuous bleeding from the site of detachment. As in the present case, patients with adenomyosis are often complicated by uterine endometriosis, which can cause extrauterine adhesions. Due to these risks, possible uterine rupture needs to be considered in pregnancies following adenomyomectomy, which may occur at any time during pregnancy. In addition, the delivery needs to be managed by a multidisciplinary team including obstetricians, pediatricians, anesthesiologists, and surgeons. Therefore, it is fundamental not to recommend pregnancy to women who underwent adenomyomectomy. However, in fact, an increasing number of women are becoming pregnant after adenomyomectomy.

The present case underwent adenomyomectomy twice before pregnancy. Furthermore, she had uterine endometriosis. The above-described risks (i.e., uterine rupture, PAS, and adhesion to the surrounding tissues) were very high. Although MRI at 22 weeks of gestation suggested imminent uterine rupture and the presence of PAS, we retrospectively considered the PAS lesion at the left posterior wall of the uterus to have formed much earlier. Persistent genital bleeding and SCH in early pregnancy also suggested this. MRI may have been performed earlier (e.g., in the early second trimester) and it is unclear whether this affected the management of this patient. Fortunately, neither intraperitoneal bleeding nor crucial rupture of the uterus occurred during pregnancy. Although pPROM and failed tocolysis forced delivery at 29 weeks of gestation, managed hospitalization allowed cesarean hysterectomy to be performed uneventfully with sufficient manpower.

Pregnancies following multiple adenomyomectomies are rare. Only two pregnancies after multiple adenomyomectomies have been reported to date, and both cases were pregnant women who underwent adenomyomectomy twice and were published in Japanese [14]. We summarized these case reports in Table 1. Both cases resulted in uterine rupture. One woman miscarried at 16 weeks of gestation and the other had a live baby at 31 weeks of gestation. Neither case was able to deliver after 34 weeks of gestation. The latter required hysterectomy due to placenta percreta, similar to the present case. On the other hand, good perinatal outcomes may be achieved following multiple adenomyomectomies that have not yet been reported when we consider a publication bias. There are currently no guidelines for the management of pregnancy following adenomyomectomy. In our institute, we admit pregnant women after adenomyomectomy to hospital for careful management after 22-25 weeks of gestation even if they are asymptomatic. If uterine contractions become frequent, tocolysis is initiated [9]. However, it remains unclear whether hospitalization with bed rest and tocolysis prolongs pregnancy and decreases the risk of an adverse maternal event.

**Table 1:** Pregnant women who underwent adenomyomectomy twice. GW: gestational weeks; IVF-ET: in vitro fertilization embryo (or blastocyst) transfer; NA: not available; PAS: placenta accreta spectrum; pPROM: preterm premature rupture of membranes

Cases	Author (year)	Age (years)	Gravida (G) and para (P)	Mode of conception	Age at 1st adenomyomectomy (years)	Age at 2nd adenomyomectomy (years)	Interval between 2nd adenomyomectomy and conception (months)	Event (GW)	Procedures	Blood loss in the procedures (mL)	Degree of PAS	Fetal or neonatal outcome	Birthweight (g)
1	Nishida (2016) [14]	38	G3P0	IVF	32	38	9	Uterine rupture (31)	Cesarean hysterectomy	NA	Increta	Live birth	NA
2	Nishida (2016) [14]	34	G1P0	Natural	31	34	NA	Uterine rupture (16)	Repair of the uterus by laparotomy	NA	Percreta	Abortion	NA
3	This case (2022)	40	G1P0	IVF	31	38	26	pPROM (29)	Cesarean hysterectomy	6060	Percreta	Live birth	1353

## Conclusions

Pregnancies following multiple adenomyomectomies are at high risk of uterine rupture and PAS. Adenomyomectomy is a good treatment option for adenomyosis and may become more widespread in women who wish for future pregnancy. A sufficient explanation associated with the risks in future pregnancy is needed, particularly after second adenomyomectomy. Although it has not yet been clarified whether bed rest and tocolysis prolong pregnancy, hospitalization may be useful for securing the condition of the patient.
